# DOG-SPOT database for comprehensive management of dog genetic research data

**DOI:** 10.1186/1751-0473-5-10

**Published:** 2010-12-15

**Authors:** Julie AS Powell, Jeremy Allen, Nathan B Sutter

**Affiliations:** 1 Office of Educational Development, College of Veterinary Medicine, Cornell University, Ithaca, 14853, USA; 2 Department of Clinical Sciences, College of Veterinary Medicine, Cornell University, Ithaca, 14853, USA

## Abstract

Research laboratories studying the genetics of companion animals have no database tools specifically designed to aid in the management of the many kinds of data that are generated, stored and analyzed. We have developed a relational database, "DOG-SPOT," to provide such a tool. Implemented in MS-Access, the database is easy to extend or customize to suit a lab's particular needs. With DOG-SPOT a lab can manage data relating to dogs, breeds, samples, biomaterials, phenotypes, owners, communications, amplicons, sequences, markers, genotypes and personnel. Such an integrated data structure helps ensure high quality data entry and makes it easy to track physical stocks of biomaterials and oligonucleotides.

## Findings

A typical genetics research laboratory is burdened by an information management challenge: to accurately store and make sense of a wide array of data of very different types [1]. A lab working with companion animals such as dogs, cats or horses needs to manage not only oligonucleotides, PCR products, markers, genes and DNA samples, but also data particular to companion animal work: owner information, pedigree relationships, registration numbers and breed names [2]. While it may be possible to track these disparate data independently, a database framework that coherently relates them and enforces lab-wide data input rules would allow for both higher overall productivity and fewer data entry errors [3]. Furthermore, such a data model would mean a single instance of the data can be easily located, backed up and computed upon.

We have developed a relational database "DOG-SPOT" (Dogs, Owners, Genotypes, Samples, Pedigrees, Oligos and Traits) implemented in Microsoft Access to provide information management for dog genetics research (additional file 1). Within a single database a laboratory can manage all of their data relating to: dogs, their breeds, phenotypes, pedigrees and kennel clubs; owners and communications with them; laboratory personnel and their activities; samples and biomaterials. Furthermore, data relating to genetics experiments can be captured, including: gene lists, oligonucleotides, amplicons and capillary sequence trace quality, in addition to genotypes for SNP, microsatellite and indel markers. See Table 1 for a list of the key database tables and fields and additional file 2 for the entity relationship diagram. Users can easily extend and customize DOG-SPOT by writing new forms, queries, macros, tables and relationships. Data quality is partially maintained by enforcing some rules about data types. For example, a dog record is required to have a non-null breed entry and the breed must be an entry in the breed table. As a second example, a dog's sire must be a dog of known sex "M" while its dam must be of known sex "F".

**Table 1:** Tables, fields, and primary and foreign keys in DOG-SPOT.

Table	Field	Primary/Foreign Key [Table]
**Activity**	id	**Primary Key**
	member_id	Member
	date	
	time_in	
	time_out	
	activity	
		

**Amplicon**	id	**Primary Key**
	F_seq	
	R_seq	
	F_Tm	
	R_Tm	
	designed_by	Member
	date_ordered	
	chr_cf2	
	start_cf2	
	end_cf2	
	gene	Gene
	purpose	
	optimization_exp	
	optimized_by	Member
	PCR_good	
	PCR_annealing_temp	
	PCR_band	
	seq_result	
	notes	
		

**Breed**	breed	**Primary Key**
	specialist	Member
	kennel_club	Kennel_Club
	notes	
	written_standard	
	collection_status	
		

**Communication**	id	**Primary Key**
	Owner_id	Owner
	action	
	action_date	
	do_next	
	by_date	
	lab_contact	Member
	completed	
		

**Dog**	id	**Primary Key**
	breed	Breed
	sex	
	owner	Owner
	registered_name	
	registration_number	
	call_name	
	date_of_birth	
	sire_id	Dog
	dam_id	Dog
	notes	
	date_of_trait_measures	
	weight	
	coat_color_pattern	
	skin_color	
	diagnosis	
	height_withers	
		

**Experiment**	id	**Primary Key**
	date	
	assay_type	
	completed_by	Member
	quality_score	
	notes	
		

**Gene**	abbreviation	**Primary Key**
	name	
	description	
	chr_cf2	
	start_cf2	
	end_cf2	
		

**Genotype**	id	**Primary Key**
	sample_id	Sample
	experiment_id	Experiment
	genotype	
	marker_id	Marker
		

**Kennel_Club**	abbreviation	**Primary Key**
	name	
	country	
	url	
	note	
		

**Marker**	id	**Primary Key**
	chr_cf2	
	start_cf2	
	end_cf2	
	allele1	
	allele2	
	left_flank	
	right_flank	
	marker_type	
	notes	
		

**Member**	netid	**Primary Key**
	first_name	
	last_name	
	active	
	description	
		

**Owner**	id	**Primary Key**
	breed_affiliation	Breed
	first_name	
	last_name	
	email	
	home_phone	
	street_address	
	city	
	state	
	zipcode	
	country	
	lab_contact	Member
	kennel_name	
	URL	
	date_entered	
	work_phone	
	cell_phone	
	fax	
	notes	
	is_contact_allowed	
	referred_by	Owner
	generate_label	
	date_of_last_mailing	
	number_kits_mailed	
	number_kits_transferred	
		

**Sample**	sid	**Primary Key**
	dog_id	Dog
	date_received	
	biomaterial_received	
	received_by	Member
	process_type	
	date_processed	
	processed_by	Member
	post_process_vol_ul	
	post_process_conc	
	post_process_OD	
	post_process_biomat	
	notes	
	source_id	Sample
		

**Sequence**	id	**Primary Key**
	name	
	amplicon_id	Amplicon
	sample_id	Sample
	experiment_id	Experiment
	sequencing_primer	
	quality_ok	
	notes	

**Figure 1:**
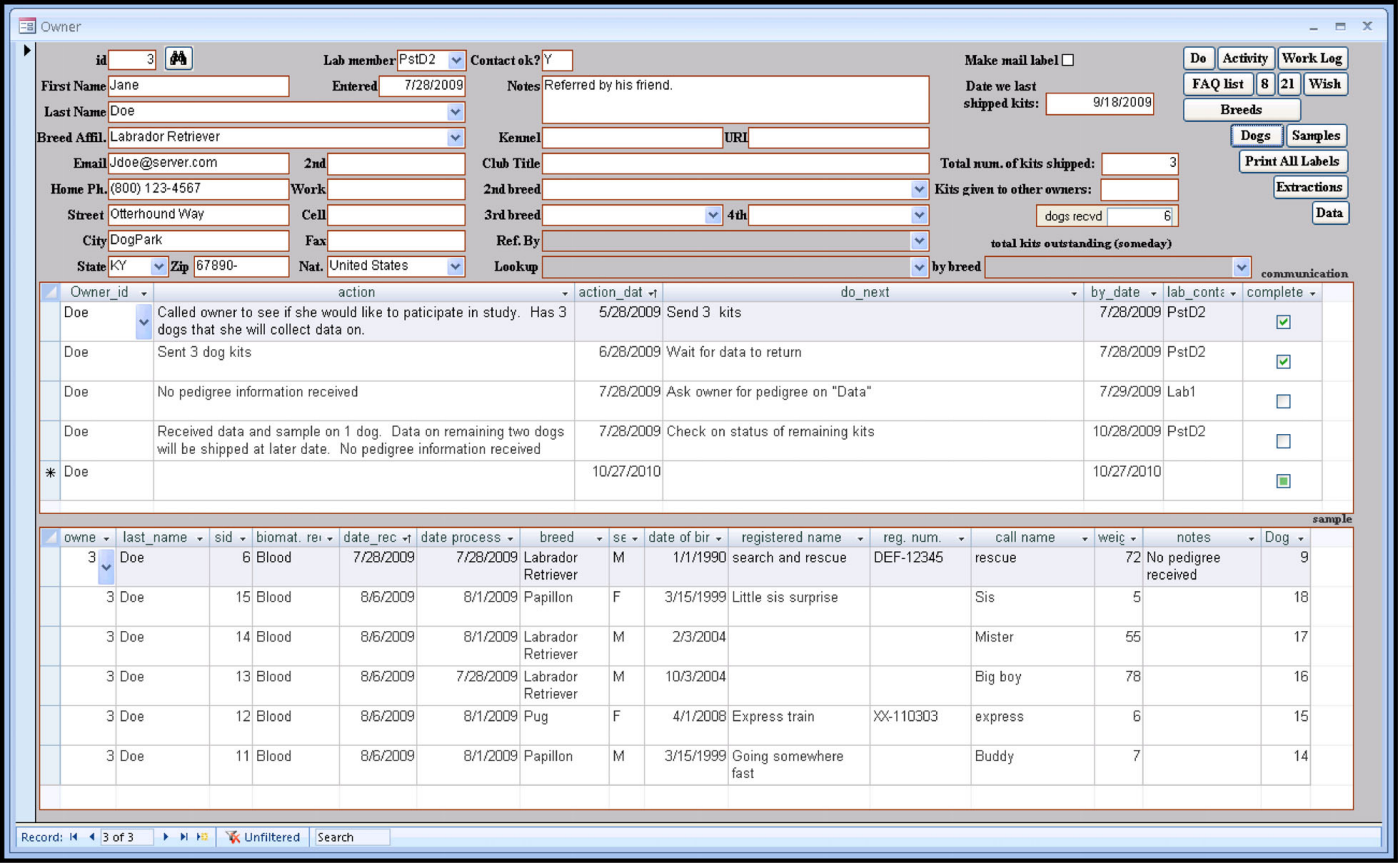
**Screen shot of the owner form**. Owner information is entered and displayed here, including a log of communications between the lab and the owner. The owner's sampled dogs are also listed. Shipment of collection kits is tracked as well, and this owner can be marked for inclusion in the next batch of printed mailing labels.

The solicitation and acquisition of high quality DNA samples, pedigrees and phenotypes from companion animals is a labor-intensive process. Often a lab needs to track a flood of correspondence between laboratory members and dozens or even hundreds of animal owners. DOG-SPOT provides an owner record for managing such relationships. Lab personnel can generate multiple communication records and reminders linked to each owner, which are displayed in an owner-centric form along with a list of dogs sampled from that owner (Figure 1). Reminders with past-due dates are filtered to an "alarm" list so they can be prioritized. Owner records can also be marked for inclusion in mailing-label print jobs. These features modularize the work flow and enable solicitation work to be efficiently handled by a team of lab members.

Within DOG-SPOT owners and dogs have a one to many relationship, as do dogs and the records of samples collected from them. The dog record itself stores registration information, breed, name, date of birth, the dam and sire IDs, as well as values for measured traits (e.g. body weight, coat color, etc.). A sample record links to exactly one dog record and stores the yield and concentration of the extracted nucleic acid, extraction date, and biomaterial type (e.g. blood, gDNA, RNA, etc.). From the dog record a three generation pedigree can be displayed as a quality check.

The 'amplicons' table stores the sequence of each forward and reverse oligonucleotide and each amplicon's chromosome, start and end position. The 'sequence' table stores a record for each sequencing reaction and a true/false value assessing overall read quality so that sequence coverage will not count as "completed" unless it is of sufficient quality. After the user aligns sequences outside the database (e.g. with phred/phrap and consed) and identifies new microsatellite, indel and single nucleotide polymorphisms, they are uploaded in batch to the 'markers' table. The markers table stores marker position start and end, chromosome, flank sequence and type. When a lab member collects data they write a record to the 'experiment' table that briefly summarizes the bench work performed. Genotype records are then uploaded from text or excel files with the experiment id, sample id, marker id and the genotype value.

Suppose, for example, that a lab member aims to collect sequence data spanning a coding exon of a gene. They design PCR primers spanning the exon and create an amplicon record in DOG-SPOT that contains the sequences of the F and R primer, the Tm of each, and the amplicon's chromosome, start and end position in the canfam2 assembly of the dog reference sequence. When the primers are synthesized and optimized the lab member would modify the record to indicate the PCR conditions to use. They then would select a set of dogs for sequencing by viewing dogs, pedigrees and DNA samples in the database's dog form. After wet-lab work and sequence alignment using, for example, phred/phrap and consed, the lab member would write a set of three text files. First, they would record which sequences were high quality. Second, they would describe every marker discovered in the sequence contig by writing the marker's flank sequences, type, alleles and position. Third, they would genotype each of the sequenced samples for each marker. These three flat files would then be uploaded/appended to the DOG-SPOT sequence, marker and genotype tables, respectively.

**Figure 2:**
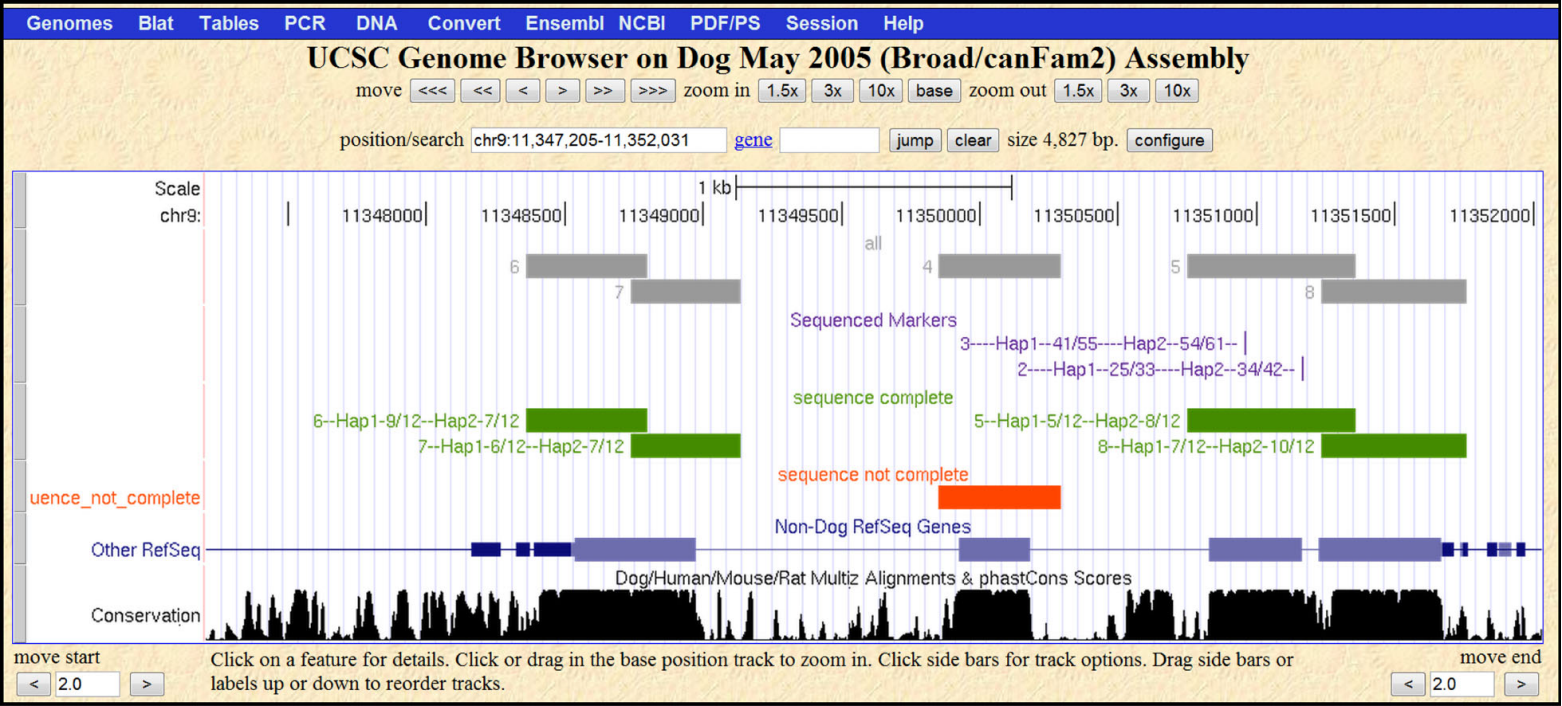
**Screen shot of the UC Santa Cruz genome browser showing uploaded DOG-SPOT amplicon and marker data**. The user runs a macro in DOG-SPOT that queries tables to generate text files of: all amplicon records (shown in gray), amplicon records for which sequence has been attempted but is poor quality or incomplete (orange), amplicon records with sufficient high quality sequence data (green) and discovered markers (purple). The user runs the *make_bed.pl *perl script in a folder with these four text files to generate the BED formatted file "bed.txt" that can then be uploaded to the genome browser. This visual overlay with UCSC tracks enables convenient assessment of candidate gene sequence coverage, completeness and the positioning of markers relative to genes.

To leverage the ease with which custom data can be uploaded and viewed in the UC Santa Cruz genome browser, DOG-SPOT stores amplicon and marker position information in a format compatible with upload: chromosome, start position and end position. To generate data for viewing in the genome browser, the user runs queries that write text files of the amplicons, sequence traces and markers. The user then executes the *make_bed.pl *perl script to generate a bed formatted file of custom data tracks that can be uploaded directly to the genome browser (see additional files 3 and 4 for the perl script and README, respectively). This visual overlay with UCSC tracks is a powerful tool for assessing the progress of sequencing through a candidate gene, for example, or to verify positioning of newly discovered markers within a gene (see Figure 2).

At present, DOG-SPOT is designed to store purebred dog records, breeds, samples and genotypic data. However, a user could readily convert it for use with cat, horse, or another species of interest. We implemented DOG-SPOT in MS-Access to provide an easy interface for lab members, some of whom had no prior database experience. However, because the database is not implemented in a more robust system, like MySQL or PostgreSQL, it would likely perform poorly if loaded with very large datasets, such as millions of array-generated genotypes.

To assist in the management of diverse types of lab data we have developed the DOG-SPOT database for the canine genetics research laboratory. By storing all of our laboratory's dog, owner, sample, amplicon, marker and genotype data in an instance of DOG-SPOT, we have successfully centrally managed these disparate data in a rational and organized way. Finally, by relying on contact management functions and modularization of work within the database, we have been able to efficiently utilize undergraduate workers for all aspects of sample solicitation, owner communication, sample data entry and biomaterial banking.

## Competing interests

The authors declare that they have no competing interests.

## Authors' contributions

JP wrote most of the visual basic code, contributed to the overall database design and reviewed the manuscript. JA extensively tested the database, contributed to design discussions and edited the manuscript. NS designed the database and wrote the manuscript. All authors read and approved the final manuscript.

## Supplementary Material

Additional file 1 **DOG-SPOT.mdb**. The DOG-SPOT database implemented in MS Access. Click here for file

Additional file 2 **Figure S1**. The entity relationship diagram for DOG-SPOT Click here for file

Additional file 3 **Make_Bed.pl**. A perl script to generate BED files. See the README for details. Click here for file

Additional file 4 **README for BED file creation from the DOG-SPOT database**. Description: A protocol for generating BED files from the database. Click here for file
