# Supplementary material for: DOG-SPOT database for comprehensive management of dog genetic research data
Source: Source Code Biol Med. 2010 Dec 15;5:10. doi: 10.1186/1751-0473-5-10 (PMC3009958; doi:10.1186/1751-0473-5-10)
Supplement: Additional file 2 — Figure S1. The entity relationship diagram for DOG-SPOT [file 1751-0473-5-10-S2.PDF]

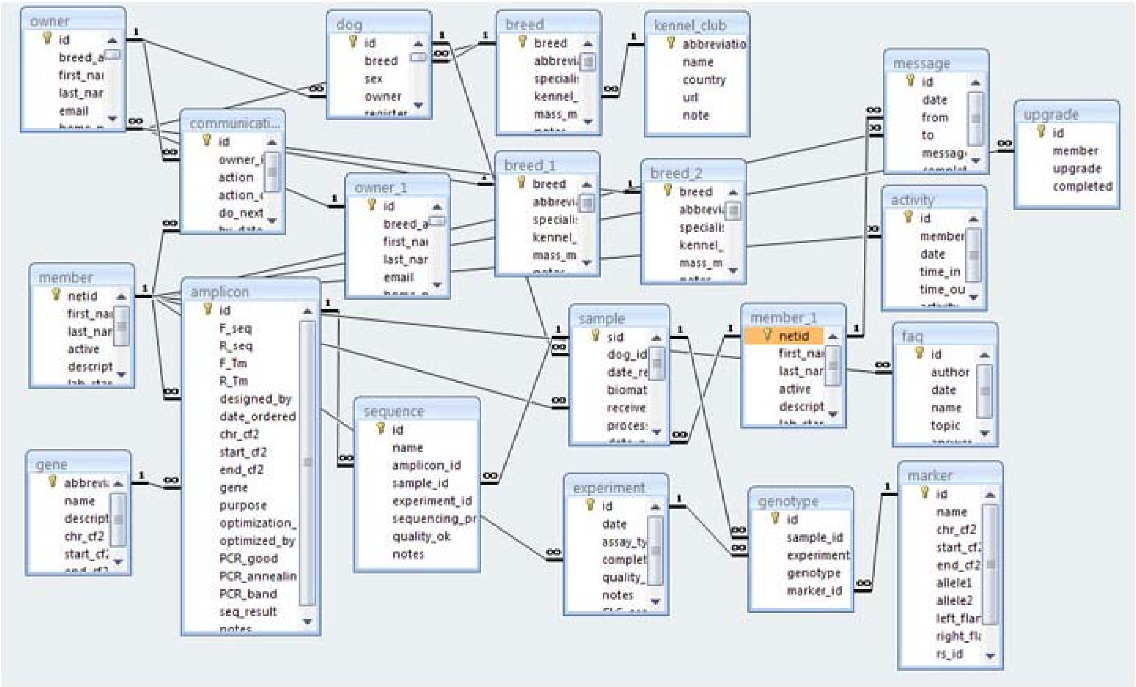

Fig. S1. An entity relationship diagram for DOG-SPOT showing tables as boxes with lists of fields. The small key graphic indicates the primary key for the table. Lines between tables indicate relationships.
