# Supplementary material for: DOG-SPOT database for comprehensive management of dog genetic research data
Source: Source Code Biol Med. 2010 Dec 15;5:10. doi: 10.1186/1751-0473-5-10 (PMC3009958; doi:10.1186/1751-0473-5-10)
Supplement: Additional file 4 — README for BED file creation from the DOG-SPOT database. Description: A protocol for generating BED files from the database. [file 1751-0473-5-10-S4.PDF]

README for BED file creation from the DOG-SPOT database.

After marker and amplicon data are uploaded to the database a browser extensible data (BED) file can be generated for upload into the UCSC genome browser. Within DOG-SPOT run the “export_BED_text_files” macro from the macros pane or by selecting the “data” button in the logon initial screen that will bring up a blue form titled “genotypes data” with a series of buttons. Select the “Make Bed File” button on the bottom of the blue form.

The macro runs a set of four queries and writes text files to a specified folder (that can be changed in the macro design view) such as \perl. Place a copy of the “Make_bed.pl” perl script in the same folder as the four text files on a machine with perl installed and run the script without arguments. On windows with perl installed to recognize the *.pl one can simply double click on the script file. A black cmd.exe window will flash and disappear and the folder will have a new file called “bed.txt”. To run the perl script if double click doesn’t work, paste a copy of the cmd.exe windows file into the same directory and double click on it to launch the dos command line. The path at the dos prompt should match the folder. At the prompt write: perl Make_Bed.txt and enter. Check for the creation of the bed.txt file.

To change colors or labels for the custom tracks open “bed.txt” in notepad or wordpad and change the R,G,B color values (range 0..255) and label string values in the header for each track.

Launch the UCSC genome browser and select “add custom tracks” from the genome browser gateway main page. Browse to the bed.txt file and upload it.
